# The Circadian Clock Drives Mast Cell Functions in Allergic Reactions

**DOI:** 10.3389/fimmu.2018.01526

**Published:** 2018-07-06

**Authors:** Pia Christ, Anna Sergeevna Sowa, Oren Froy, Axel Lorentz

**Affiliations:** ^1^Institute for Nutritional Medicine, University of Hohenheim, Stuttgart, Germany; ^2^Food Science and Nutrition, the Robert H. Smith Faculty of Agriculture, Food and Environment, Institute of Biochemistry, The Hebrew University, Rehovot, Israel

**Keywords:** mast cells, biological clock, circadian rhythm, allergy, IgE

## Abstract

Allergic diseases are known to vary in the severity of their symptoms throughout the day/night cycle. This rhythmicity is also observed in mast cell function and responsiveness. Mast cells are key effector cells of allergic reactions and release cytokines, chemokines, and important inflammatory mediators such as histamine, which have been shown to display diurnal variation. Recent research clarified that mast cells are controlled by their internal clock—which is regulated by a specific set of clock genes—as well as external factors such as light sensed by the suprachiasmatic nuclei, hormonal status, or diet. Here, we give an overview of the connections between circadian clock, mast cells, and allergic disease. Further work aimed at studying the role of chronotherapy/chronomedicine should take into account this rhythmic nature of not only mast cells but also the immune responses generated by mast cell signaling.

## Introduction

The human biological clock is a remarkable built-in mechanism, which works according to the Earth’s rotation and to changes in light, temperature, and environment, to create a 24-h rhythm. This rhythm is orchestrated by a central pacemaker in the brain that resets the clocks in peripheral organs and tissues to control the expression of key genes throughout the day. The circadian clock, driven by cell-autonomous biological clocks (1, 2), takes its cues from cycles of light and dark but also from levels of hormones or metabolic status such as feeding to directly modulate peripheral clocks, thereby affecting many physiological processes, which include response to medications or immunity (3–6). A multitude of immunological processes have been shown to be linked to the biological clock or to function under the control of circadian rhythms. Steroid levels, for example, are known to naturally cycle with the circadian clock whereby the feedback loop, which controls the release of cortisol is most apt to respond to synthetic corticosteroid treatment in the morning rather than at other times of day giving way to the inclusion of timing in the treatment of various conditions, a term known as chronotherapy or chronomedicine (7). Chronomedicine has taken a boost in interest as the Nobel Prize for Physiology or Medicine was recently awarded to Jeffrey Hall, Michael Rosbash, and Michael Young who elucidated the cellular mechanism behind circadian rhythms (8). Now it is known that many immunological processes including the number of immune cells, cytokines, and chemokines differ throughout the day with peak times dependent on tissue and circadian gene expression (9) leading to a connection between the biological clock and levels of inflammation, development of disease, and response to treatment (10, 11).

**Figure 1:**
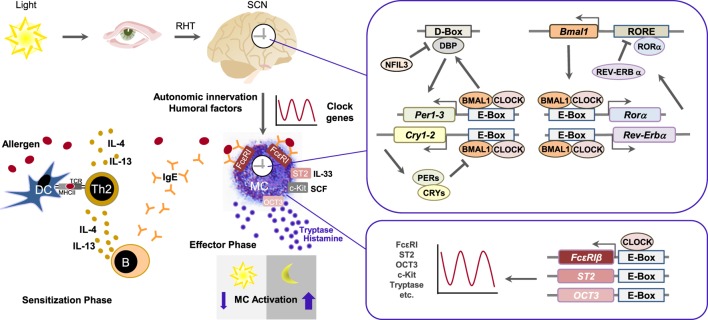
The mast cell clock in type I allergic reaction. Allergens are taken up by antigen-presenting cells such as dendritic cells (DC), which present them to naïve CD4^+^ T cells *via* major histocompatibility complex class II. In the presence of IL-4, naïve CD4^+^ T cells differentiate into Th2 cells. Secretion of IL-4 or IL-13 by Th2 cells causes an isotype switch to IgE in B cells. Allergen-specific IgE engages to FcεRI on mast cells. If allergens bind to specific IgE, FcεRI is cross-linked, followed by the release of mast cell mediators such as histamine and tryptase and induction of allergic symptoms. The circadian clock consists of the central oscillator, located in the suprachiasmatic nucleus (SCN) of the hypothalamus, and peripheral oscillators present in virtually all cell types. Light activates photoreceptors and *via* retinohypothalamic tract (RHT) the central SCN clock. Peripheral circadian clocks are synchronized and entrained by autonomic innervation and humoral factors. Clocks in peripheral tissues use the same molecular components like present in SCN, as the core molecular clock consists of interlocked transcriptional and translational feedback loops. Core clock proteins BMAL1 and CLOCK form a heterodimer and, by binding to E-box-motifs, induce the expression of other clock components. Among them are BMAL1 positively regulatory proteins like RORα as well as negatively regulatory proteins like REV-ERBs, PERs, and CRYs, thus concurrently attenuate their own transcription and initiate a new transcription cycle. DBP and NFIL3 form another loop that regulates transcription of genes containing D-box sequences, including those for PERs, and thus cooperate with the core clock to establish robust 24-h rhythms. The mast cell clock may temporally gate expression of FcεRI, ST2, OCT3, etc., *via* CLOCK and E-box elements, thereby generating a marked circadian variation in IgE/mast cell-mediated allergic reactions.

Allergic conditions such as asthma or allergic rhinitis have historically shown circadian bias as the severity of symptoms is exacerbated between midnight and morning time and exhibits prominent 24-h variation (12, 13). Mast cells are known to have multiple immunoregulatory functions through the release of their mediators, such as histamine, leukotrienes, cytokines, chemokines and proteases and also serve as the key drivers of long-term pathophysiological changes associated with chronic allergic responses (14, 15). Mast cell activation occurs during type I allergic reactions *via* antigen-mediated aggregation of immunoglobulin E-bound FcεRIs (Figure 1) (16, 17). As they are the main effector cells in allergy, the rhythmicity of mast cells has also come under investigation. For example, in some of the earlier findings, serum mast cell tryptase and plasma histamine levels were shown to be lower in the afternoon but peaked during night (18, 19). In the following review, we give a brief overview of the role of the circadian clock in regulating mast cells and allergic reactions. As there are various types of mast cells (20), it is conceivable to think that the circadian clock regulates the expression of type-specific genes leading to different functions.

## The Molecular Mechanism of the Circadian Clock

In mammals, the core molecular clock consists of interlocked transcriptional and translational feedback loops that regulate the expression of clock genes (Figure 1). The transcription factors *brain and muscle ARNT-like protein 1* (BMAL1) and *circadian locomotor output cycles Kaput* (CLOCK) form a heterodimer and induce the transcription of other clock genes *via* binding to E-box-motifs (5′-CANNTG-3′). Among the transcriptional output are BMAL1 positively regulatory proteins like *RAR-related orphan receptor alpha* (RORα), as well as negatively regulatory proteins like *REV-ERBs* (REV-ERBα, REV-ERBβ), *Periods 1-3* (PER1, PER2, PER3), and *Cryptochromes* (CRY1, CRY2). RORα and REV-ERBα/β act by binding to the ROR response element (RORE) of target genes. Upon oligomerization, PER and CRY inhibit BMAL1/CLOCK dimers and concurrently attenuate their own transcription, thus initiating a new transcription cycle. Albumin D-box binding protein (DBP) and the repressor nuclear factor interleukin 3 establish another loop that regulates transcription of genes containing D-box sequences, including those for PERs, and thus contribute to set robust 24-h rhythms. Furthermore, posttranslational modifications as well as other secondary clock proteins that feedback to the core clock mechanism add to the complexity of this molecular network (2, 21–24).

## The Circadian Clock in Mast Cells

It has previously been demonstrated that mast cells have a functional molecular clock and that the core clock genes show rhythmic circadian expression patterns (25, 26). Murine clock genes *mClock, mBmal1, mPer1*, and *mPer2*, as well as mast cell-specific genes *mMcpt-5, mMcpt-7, mc-kit*, and *mFc*ε*RI* α*chain* (and the proteins mMCPT5 and mc-Kit) showed circadian mRNA expression in mast cells isolated from mouse jejunum (25). Murine bone marrow-derived mast cells (BMMCs) demonstrated circadian expression of *mPer1, mPer2, mBmal1, mRev-erb*α, and *mDbp* (26) and human intestinal mast cells also exhibited circadian regulation of the expression of *hPer1, hPer2*, and *hBmal1* as well as *hTryptase* and *hFc*ε*RI* (25).

As the IgE/mast cell axis plays a central role in allergic diseases, including allergic rhinitis, asthma, urticaria, and food allergy, further work on the role of IgE stimulation is important to understand the cellular underpinnings of these events (15, 27). Experiments of our group showed that human intestinal mast cells produce a circadian release of *de novo* synthesized cysteinyl leukotrienes and pre-stored histamine upon IgE-mediated activation (25, 26). Moreover, stimulation at different time points *via* FcεRI resulted in rhythmic interleukin (IL)-13 and IL-6 cytokine mRNA expression in BMMCs. FcεRI α chain mRNA and protein levels displayed a circadian pattern, which could explain the circadian oscillation of cytokine production in response to activation *via* FcεRI (26). We subsequently found that the phosphorylation of the signaling molecule extracellular-signal regulated kinase 1/2 (ERK1/2) in response to FcεRI crosslinking showed circadian rhythms (28). ERK1/2 plays a major role in cytokine expression, degranulation, and arachidonic acid metabolism in mast cells (29). Thus, the clock affects activation of signaling molecules, such as ERK1/2, and thereby the functionality of mast cells resulting in circadian production and release of mediators. The impact of the clock on the reactivity of mast cells may explain how mast cells can contribute to the circadian variation of allergic symptoms. Noteworthy, cells in culture are reset by dexamethasone treatment, medium change, or serum shock. These treatments could affect several signaling as well as metabolic pathways, which might also interfere with the analyses (30, 31).

Different mouse models can further elucidate these roles. Wild-type mice displayed variations in allergic reactions depending on time of day while this variation was lacking in *Per2*- and *Clock*-mutated mice (32). Using mice with a loss-of-function mutation of Per2 (mPer2^m/m^ mice), Nakamura et al. identified PER2 as a regulator of time of day-dependent variation in passive cutaneous anaphylactic reaction. The time-of-day-dependent variation observed in wild-type mice was absent in mPer2^m/m^ mice. Moreover, loss of rhythmic secretion of corticosterone was obtained in mPer2^m/m^ mice, accompanied by a decreased sensitivity to dexamethasone. Interestingly, there was no difference in IgE-mediated degranulation between BMMCs of wild-type and mPer2^m/m^ mice, as PER2 possibly regulates the response to glucocorticoids in mast cells. However, *Per2* mutations affect all cells and, therefore, it could not be identified, whether it is PER2s’ control of adrenal glands and glucocorticoid secretion and/or its impact on mast cell clock that is causative for time-of-day-dependent variations in passive cutaneous anaphylactic reaction (33).

The important role of the mast cell-intrinsic clockwork in IgE/mast cell-mediated allergic reactions is supported by the finding that the time-of-day-dependent variation of cutaneous anaphylactic reaction was also absent in mice with a *Clock* mutation in mast cells. Using mast cell-deficient W/Wv mice subcutaneously reconstituted with BMMCs generated from wild-type mice and mice with a dominant negative-type mutation of *Clock* (Clock^D19/D19^ mice), Nakamura et al. reported that this *Clock* mutation was accompanied by the loss of temporal regulation of FcεRI expression, signaling, and the absence of variations in IgE-mediated degranulation *in vivo* and *vitro* (34). They observed circadian mRNA expression of FcεRI β, the β subunit of FcεRI, which has been shown to function as an amplifier of the high-affinity IgE receptor (35). However, they did not detect circadian expression of other FcεRI signaling–associated molecules upstream of Ca2^+^ signaling, such as FcεRI α and FcεRI γ, Syk, and STIM1 whereas Wang et al. and we found circadian expression of FcεRI α (25, 26). Several E-box–like elements to which the CLOCK/BMAL1 complex can theoretically bind are present in the promoter region of the mouse FcεRI β chain; therefore, the authors further tested whether CLOCK binds to the promoter region of FcεRI β. They observed that the transcription of FcεRI is under control of the mast cell clock as CLOCK could be shown to bind to the promoter of FcεRI β chain (34). Table 1 summarizes clock genes analyzed in mast cells and depicts there circadian impact on mast cells.

**Table 1:** Clock genes analyzed in mast cells.

Clock gene	Regulated *via*	Activator*via*	Repressor*via*	Mast cell-related function
*Bmal1*	RORE	E-Box		Shows circadian expression in mast cells [BMMC (26), hiMC (25), mjMC (25)]

*Clock*	RORE	E-Box		Shows circadian expression in mast cells [BMMC (26), hiMC (25), mjMC (25)]; affects IgE levels (36); binds to promotors of mast cell-related genes [*Fc*ε*RI* β *chain* (34), *Oct* (37), *St2* (38)] *via* E-Box

*Cry1/2*	E-Box		E-Box	Shows circadian expression in mast cells [BMMC (26), hiMC (25), mjMC (25)]
	D-Box			
	RORE			

*Dbp*	E-Box	D-Box		Shows circadian expression in mast cells [BMMC (26)]

*Per1/2*	E-Box		E-Box	Shows circadian expression in mast cells [BMMC (26), hiMC (25), mjMC (25)]; affected by cortisone levels (*Per2*) (39); may regulate response of mast cell to glucocorticoids (39); involved in resetting the clock (*Per2*) (39); necessary for time-of-day-dependent variation in passive cutaneous anaphylactic reaction (*Per2*) (33)
	D-Box			

*Rev-erb*α	E-Box		RORE	Shows circadian expression in mast cells [BMMC (26)]
	D-Box			

Intrinsic mast cell clock (not yet specified)				Circadian expression of FcεRI α chain mRNA [BMMC (26), hiMC (25)] and protein [BMMC (26)], mMCPT5, and mc-Kit [mjMC (25)], IL-13 and IL-6 mRNA [BMMCs (26)], tryptase [hiMC (25)]; circadian expression and release of CXCL8, CCL2 [hiMC (28)], release of histamine and leukotrienes [hiMC (25)], and phosphorylation of ERK1/2 [hiMC (28)] in response to activation *via* FcεRI

*The transcriptional circuit underlying the circadian nature of mast cells consists of the clock-controlled elements E-Box, D-box, and RORE with many clock-controlled genes. Listed are clock genes that are analyzed in mast cells and are related to circadian function in mast cells. Clock genes are given with their relevant regulatory elements and whether the clock protein acts as a transcriptional activator or transcriptional repressor *via* either binding to E-box, D-box, or RORE elements (40–45). hiMC, human intestinal mast cells, mjMC, mouse jejunal mast cells; BMMC, mouse bone marrow-derived mast cells; RORE, ROR response element*.

## Mechanisms Underlying the Circadian Function of Mast Cells

The molecular mechanisms underlying the circadian function of mast cells are still under investigation but core transcription factors of the molecular clock (i.e., CLOCK) together with common regulator sequences such as the E-box elements seem to be critical for this mechanisms (Figure 1). Downstream targets include mast cell mediators such as cytokines and histamine release, both of which in turn can serve as modulators of the circadian clock. For example, pro-inflammatory cytokines, such as tumor necrosis factor (TNF)-α or IL-1β, known to be expressed by mast cells (46), have been shown to be involved in altered expression of clock genes in cultured fibroblasts (30). TNF-α has also been found to have an impact on the expression of core clock genes in mice (47). IL-33 is a cytokine that executes diverse functions in innate and acquired immune responses, including allergic reactions (48). It strongly stimulates innate immune cells such as mast cells, basophils, and group-2 innate lymphoid cells to produce various cytokines/chemokines, including IL-6, IL-13, and TNF-α *via* the IL-1 receptor-like 1 protein ST2 (49). Kawauchi et al. observed that, similar to IgE/mast cell-mediated allergic reactions and expression of FcεRI β, CLOCK also temporally regulates mast cell response to IL-33 that was absent in *Clock*-mutated mice by directly binding to the promotor region of ST2 and thus inducing ST2 expression. Thereby, CLOCK affects time-of-day-dependent cytokine release of IL-6, IL-13, and TNF-α in wild-type BMMCs, as well as IL-13 and Gob-5 mRNA expression, and neutrophil infiltration (38).

Histamine levels are another potent downstream target. Allergic symptoms exacerbate during nighttime and plasma histamine levels exhibit nocturnal peaks. In mastocytosis patients, peak levels of plasma histamine were observed in the early morning with the lowest in the afternoon (19). Interestingly, circadian variations of plasma histamine levels were diminished in mast cell-deficient mice reconstituted with *Clock*-mutated BMMCs demonstrating the influence of the mast cell clock (37). Organic cation transporter 3 (OCT3), which is involved in histamine transport, is temporally controlled by *Clock* in mast cells by binding to the promoter of mouse OCT3. Circadian variations in plasma histamine levels were lost upon inhibition of OCT3. Stress caused desynchronization of the mast cell clock, which was associated with the loss of circadian variations in OCT3 expression and thereby plasma histamine levels (37). Thus, stress can disrupt the proper role of mast cells in regulating histamine levels, which in turn serve as modulators of circadian rhythm.

Histamine release also connects mast cells, the biological clock, and the hypothalamus. Intracranial mast cells of dogs passively sensitized with IgE and subsequently antigen-treated caused a marked increase in cortisol secretion of the adrenal gland, whereas pretreatment with histamine H1 blocker or anti-corticotropin-releasing factor antibodies, attenuated the increasing cortisol level. This demonstrates the influence of brain mast cells on hypothalamic–pituitary–adrenal axis (50), which can be activated by anaphylactic shock or administration of histamine (51, 52). Additionally, histamine was shown to inhibit dopamine release in the mouse striatum by modulating presynaptic H3 receptors (53). These studies implicate a possible contribution of mast cells to the regulation of the systemic clock by histamine release.

## Chronoregulation of Mast Cells

While mast cells have downstream mediators, which can account for the rhythmicity of certain conditions, they are themselves under the control of various biological functions. One of these functions is nutritional status. Restricted food intake and base energy consumption of an organism can reset the circadian clock, alleviate inflammation, and have been suggested to be beneficial for health (54, 55). We thus analyzed relative mRNA of mast cell-specific markers, including mast cell protease 4 (MCPT-4), mast cell chymase1 (MCPT-5/CMA1), and c-Kit receptor (C-KIT/CD117) for circadian expression in mice jejunum under various feeding conditions. Under conditions of *ad libitum* food intake, *mMcpt-4* and *mMcpt-5* displayed an oscillatory pattern while during time-restricted feeding, they expressed a higher amplitude, showed a phase advance, and became more robust although the daily average level wasn’t increased significantly. Interestingly, an adaptation to food intake time could be detected, as *mMcpt-4* peaked just before food was given under restricted feeding conditions (55). These findings highlight a role of nutrition in regulating peripheral clocks in tissues and cells.

Indirectly responsible for the timing of food intake is the principal environmental stimulus for circadian rhythms in mammals, the light pattern based on 24 h light–dark cycles (5). Initiated by light activating intrinsically photosensitive ganglion cells in the retina, the suprachiasmatic nucleus (SCN) of the anterior hypothalamus is entrained *via* the retinohypothalamic tract (RHT). The SCN, region of the circadian pacemaker, in turn synchronizes peripheral clocks by using mediators such as neuronal signals and hormones (56). Hormones and neurotransmitters released by autonomic nervous system and the hypothalamus–pituitary–adrenal gland axis control and modulate clocks and functions of various cell types, including mast cells.

Mechanical disruption of the SCN resulted in the absence of a time of day-dependent variation in passive systemic anaphylactic reaction in mice, accompanied by the loss of circadian variations in serum histamine, MCP-1 (CCL2), and IL-6 levels (57). Mast cell clocks may also be controlled by rhythmic secretion of glucocorticoids of the adrenal glands as the rhythmic response to IgE-dependent activation in a mouse model of passive cutaneous anaphylaxis was lost not only in *Per2*-mutant mice but also in adrenalectomized mice, associated with an aberrant daily level of serum corticosterone. If serum corticosterone level was high during day-time, *Per2* expression in mast cells was high as well, indicating the influence of the systemic clock on mast cells (33). It is well known that plasma concentrations of adrenocorticotrophic hormone and cortisol show circadian variations in humans (58), controlled by SCN and adrenal glands (59), and that glucocorticoids are potent in resetting circadian clocks. Inhalation of dexamethasone—a glucocorticoid receptor agonist—caused a 6-h phase advance in gene expression of *mPer1, mPer2*, and *mClca3* in lungs of ovalbumin-treated and control mice (60). Stability of core clock proteins is determined by posttranslational modifications. Casein kinases CK1δ and CK1ε play a pivotal role as they phosphorylate PER proteins, leading to their degradation (61). Corticosterone or treatment with PF670462, a selective inhibitor of casein kinase 1d/ε, averted IgE-mediated allergic reactions *in vitro* and *vivo*. Passive cutaneous anaphylactic reactions exhibited increased levels of PER2 in mast cells that were ameliorated by PF670462 or corticosterone treatment of mice (39).

## Concluding Remarks

The circadian clock modulates a multitude of human conditions including asthma and allergy although the cellular mechanisms regulating the clock are still under investigation. Mast cells, which serve as key effector cells in allergic disease were shown to be under control of the SCN and to have a circadian expression and release of their mediators in response to activation. Being ubiquitously distributed throughout connective and mucosal tissues and near blood vessels, mast cells are uniquely able to affect other immune cells and serve as a transition point from innate to adaptive immune response (46, 62). Hence, disrupted mast cell clocks could impair the subsequent adaptive immune responses and trigger or fortify allergic symptoms.

As there is growing evidence on the importance of the biological clock in allergic syndromes, targeting mast cell clock can be considered a valuable target of chronotherapy. Resetting the mast cell clock has shown promise in patients responding to subcutaneous allergen immunotherapy as well as to artificial glucocorticoids for asthma treatment (11, 63, 64). The biological clock is quickly becoming another lever in the field of personalized medicine, which aims to add the time factor as another dimension of therapy. Getting a better understanding of the role of mast cells, and other effector cells, in their circadian responses can help people live better lives just by changing the timing or scheduling of medications. Although practical challenges remain, the goal of uncovering the timing of mast cell signaling holds great promise for future medical applications and the understanding of human health and disease.

## Author Contributions

AL, PC, and AS designed the manuscript and were involved in drafting. OF revised the manuscript. All authors read, corrected, and approved the final manuscript. Figure was made by AL.

## Conflict of Interest Statement

The authors declare that the research was conducted in the absence of any commercial or financial relationships that could be construed as a potential conflict of interest.
